# Imbalance of Circulatory T Follicular Helper and T Follicular Regulatory Cells in Patients with ANCA-Associated Vasculitis

**DOI:** 10.1155/2019/8421479

**Published:** 2019-12-02

**Authors:** Ying Xu, Hongmei Xu, Yu Zhen, Xueting Sang, Hao Wu, Cong Hu, Zhanchuan Ma, Miaomiao Yu, Huanfa Yi

**Affiliations:** ^1^Central Laboratory of the Eastern Division, The First Hospital of Jilin University, Changchun, Jilin 130021, China; ^2^Urology Center, The First Hospital of Jilin University, Changchun, Jilin 130021, China; ^3^Department of Obstetrics, The First Hospital of Jilin University, Changchun, Jilin 130021, China; ^4^Department of Dermatology, The First Hospital of Jilin University, Changchun, Jilin 130021, China; ^5^Center for Reproductive Medicine & Prenatal Diagnosis, The First Hospital of Jilin University, Changchun, Jilin 130021, China

## Abstract

Antineutrophil cytoplasmic antibody- (ANCA-) associated vasculitis (AAV) is characterized by small-vessel inflammation in association with autoantibodies. Balance between T follicular helper (Tfh) cells and T follicular regulatory (Tfr) cells is critical for humoral immune responses. Accumulating evidence supports that Tfh and Tfr are involved in autoimmune diseases; however, their roles in AAV are unclear. In this study, we tested the changes of circulatory Tfh and Tfr in patients with AAV. Twenty patients with AAV and twenty healthy controls were enrolled. Sixteen AAV patients had kidney involvement. We found that the AAV patients had increased circulating Tfh cells (CD4^+^CXCR5^+^CD25^−^CD127^interm-hi^), decreased Tfr cells (CD4^+^CXCR5^+^CD25^+^CD127^lo-interm^), and elevated Tfh/Tfr ratios compared with healthy controls (*P* < 0.01). The Tfh percentage and Tfh/Tfr ratio, but not Tfr percentage, were positively correlated to proteinuria levels and BVAS scores in patients with AAV (*P* < 0.01). In addition, AAV patients had decreased circulating Tfh1 (CCR6^−^CXCR3^+^), but increased Tfh2 cells (CCR6^−^CXCR3^−^), compared with healthy controls (*P* < 0.01), indicating a Tfh1-to-Tfh2 shift. Furthermore, remission achieved by immunosuppressive treatment markedly attenuated the increase of total Tfh (*P* < 0.01) and Tfh2 cells (*P* < 0.05), promoted the Tfh1 response (*P* < 0.05), and recovered the balance between Tfh/Tfr cells (*P* < 0.05) and between Tfh1/Tfh2 cells (*P* < 0.05) in patients with AAV. Plasma levels of IL-21, a cytokine secreted by Tfh cells, were elevated in AAV patients compared with healthy controls (*P* < 0.01), which was attenuated by immunosuppressive treatment (*P* < 0.05). Taken together, our findings indicate that circulatory Tfh/Tfr ratios, Tfh2/Tfh1 shift, and plasma IL-21 levels are associated with AAV and disease activity.

## 1. Introduction

Antineutrophil cytoplasmic antibody- (ANCA-) associated vasculitis (AAV) is a group of potentially life-threatening autoimmune diseases in which the kidney is frequently involved [1]. Pathogenesis of AAV is not fully understood by far [2]. Majority of patients with AAV can achieve temporary disease remission with immunosuppressive induction therapy. However, acute relapses occur in more than 50% of patients during follow-up [3]. Therefore, prediction of AAV relapse has become a critical and unsolved topic. Biomarkers that are correlated with the disease activity of AAV should be useful for relapse prediction; however, they are still unavailable [4]. ANCA are autoantibodies that are abnormally produced to target and attack cytoplasmic granules of neutrophils and monocytes, leading to necrotizing vascular inflammation [5]. Although ANCA has been considered the initiator of AAV and used for AAV diagnosis for decades, ANCA titers are not associated with disease activity and are not able to predict relapse in patients with AAV [6–8]. Thus, it is important to investigate novel biomarker candidates that are associated with ANCA and disease activity.

Although production of ANCA autoantibodies is naturally dependent on B cells, certain subsets of T cells play a regulatory role in B cell response and autoantibody synthesis [9, 10]. T follicular helper (Tfh) cells promote germinal center formation and support B cell producing antibodies, while T follicular regulatory (Tfr) cells suppress antibody production [10, 11]. Both Tfh and Tfr cells are subsets of CXCR5^+^CD4^+^ T cells. One of their distinctions is that Tfh cells are CD25^−^CD127^interm-hi^ whereas Tfr cells are CD25^+^CD127^lo-interm^. As Tfh and Tfr cells have opposing roles in regulating humoral immune responses, their balance is important for immune homeostasis [9, 11, 12]. Imbalance between Tfh and Tfr cells promotes defective antibody production and contributes to the development of autoimmunity [13–15]. Recently, Tfh cells are subdivided into Tfh1 (CCR6^−^ CXCR3^+^) and Tfh2 (CCR6^−^ CXCR3^−^) subtypes, and the Tfh1/Tfh2 ratio can be altered under certain pathological conditions. However, the roles of Tfh/Tfr balance and Tfh2/Tfh1 shift in AAV are unclear.

Following immune responses, a small number of Tfh and Tfr cells from lymph nodes are released into the circulation. Circulatory Tfh and Tfr cells serve like memory cells and are able to react quickly during subsequent immune responses [16]. In addition, circulatory Tfh and Tfr cells are convenient to test, and their counts could represent the balance between Tfh and Tfr cells [17]. In this study, we investigated the relationship between Tfh/Tfr balance and disease activity in patients with AAV. We found that, compared with healthy controls, AAV patients had increased circulatory Tfh/Tfr and Tfh2/Tfh1 ratios, which were attenuated during disease remission.

## 2. Materials and Methods

### 2.1. Patients

The study was approved by the ethical committee of the First Hospital of Jilin University. Written informed consent was obtained from all individual participants. We enrolled twenty AAV patients with kidney involvement and twenty age-matched healthy controls from the outpatients and inpatients in the First Hospital of Jilin University during July 2016 to June 2017. Demographic, clinical, and laboratory parameters of the patients and healthy controls were collected. Remission was achieved by treatment with a combination of glucocorticoids and cyclophosphamide following the current guideline for clinical practice [18]. AAV is defined as necrotizing vasculitis associated with ANCA and was diagnosed according to the 1994 Chapel Hill Consensus Conference Criteria for Vasculitis [19]. Disease activity of AAV was assessed using the Birmingham Vasculitis Activity Score (BVAS) system. A BVAS of >0 was considered to represent active disease, while BVAS = 0 was defined as remission [19]. The AAV subtypes of microscopic polyangiitis and granulomatosis with polyangiitis were defined based on the Chapel Hill Consensus Criteria [20]. Kidney involvement was defined by a kidney biopsy showing pauci-immune glomerulonephritis or active urine sediment with or without worsening renal dysfunction along with a diagnostic biopsy result of small-vessel necrotizing vasculitis in an extrarenal tissue [21]. Lung complications were defined as described previously [22].

### 2.2. Flow Cytometry

Fresh venous blood samples were collected from healthy controls and the patients with AAV at the time of diagnosis and when the patients achieved disease remission. Peripheral blood mononuclear cells were isolated by density-gradient centrifugation using the Ficoll-Paque. The isolated cells were stained with the following antibodies: Alexa Fluor 700-conjugated anti-CD4 (Cat#557922, BD Biosciences, San Jose, CA, USA), PerCP-Cy5.5-conjugated CXCR5 (Cat#562781, BD Biosciences), Horizon V500-conjugated CD127 (Cat#563086, BD Biosciences), Alexa Fluor 647-conjugated anti-CD25 (Cat#563598, BD Biosciences), APC-Cy7-conjugated CXCR3 (Cat#353721, BioLegend, San Diego, CA, USA), Alexa Fluor 488-conjugated anti-forkhead box P3 (FOXP3, Cat#561181, BD Biosciences), and PE-Cy7-conjugated CCR6 (Cat#560620, BD Biosciences). Flow cytometry was analyzed with an LSR II instrument (BD Biosciences). The data were analyzed with FlowJo software (Tree Star, Ashland, OR, USA).

### 2.3. Enzyme-Linked Immunosorbent Assays (ELISA)

Plasma was collected from the whole blood. Plasma interleukin-21 (IL-21) concentrations were measured by an ELISA kit (Abcam, Cambridge, MA, USA).

### 2.4. Statistical Analysis

Statistical analysis was performed with GraphPad Prism 6.0 software (GraphPad Software Inc., San Diego, CA, USA). Normally distributed continuous variables were presented as the mean ± standard deviation and analyzed by Student's *t*-test for comparing two groups or one-way ANOVA with the post hoc Tukey honestly significant difference test for comparing multiple groups. Pearson's correlation and linear regression were performed to examine the relationship between two parameters. Differences were considered significant at a probability level of *P* < 0.05.

## 3. Results

### 3.1. Patient Characteristics

The demographic, clinical, and laboratory characteristics of the patients with AAV and healthy controls are shown in Table 1. Sixteen of the twenty AAV patients had kidney involvement and dysfunction (Table 1).

### 3.2. Increased Circulatory Tfh but Decreased Tfr Cells in Patients with AAV

**Figure 1:**
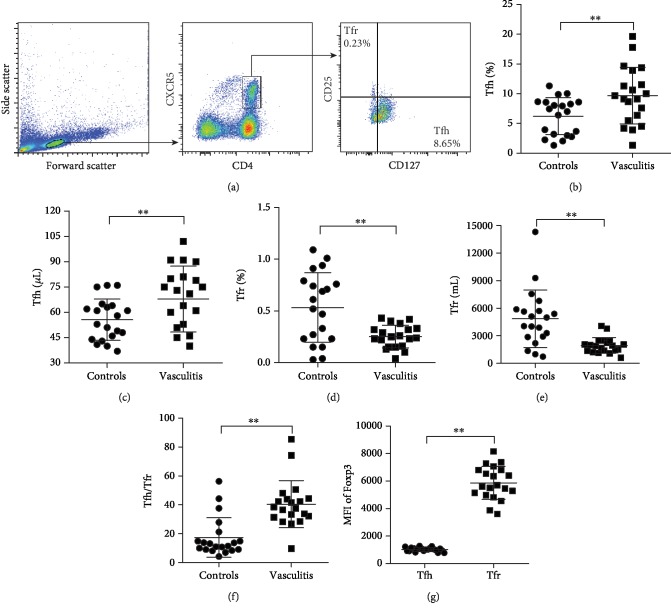
Circulatory Tfh and Tfr cells in patients with antineutrophil cytoplasmic antibody- (ANCA-) associated vasculitis (AAV). (a) The gate strategy for circulatory Tfh and Tfr cells. The percentage and count of circulatory Tfh (b, c) and Tfr (d, e) cells and Tfh/Tfr ratios (f) in patients with AAV and healthy controls. (g) Mean fluorescence intensity (MFI) of FOXP3 in Tfh and Tfr cells. ^∗∗^*P* < 0.01. Tfh: T follicular helper cells; Tfr: T follicular regulatory cells.

Circulatory CD4^+^ T cell percentages were similar between patients with AAV and healthy controls (51.49 ± 12.49%*vs.*46.21 ± 14.89%, *P* > 0.05). Circulatory Tfh and Tfr cells were analyzed using a previously reported gating strategy [17].  Figure 1(a) demonstrates circulating Tfh (CD4^+^CXCR5^+^CD25^−^CD127^interm-hi^) and Tfr (CD4^+^CXCR5^+^CD25^+^CD127^lo-interm^) cell populations that were defined by the expression of CD4 and CXCR5 and further separated by the expression of CD25 and CD127. Patients with AAV had a higher number of circulatory Tfh cells (Figures 1(b) and 1(c)) while less circulatory Tfr cells (Figures 1(d) and 1(e)) than healthy controls (all *P* < 0.01), and the Tfh/Tfr ratio was increased in patients with AAV compared to healthy controls (*P* < 0.01, Figure 1(f)). Expression of FOXP3, a T regulatory (Treg) cell marker was abundant in Tfr cells but was much less and minimal in Tfh cells (*P* < 0.01, Figure 1(g)). These results indicate that patients with AAV have an imbalance between circulatory Tfh and Tfr cell populations.

### 3.3. Tfh/Tfr Ratios Correlate with Disease Activity and Renal Injury

**Figure 2:**
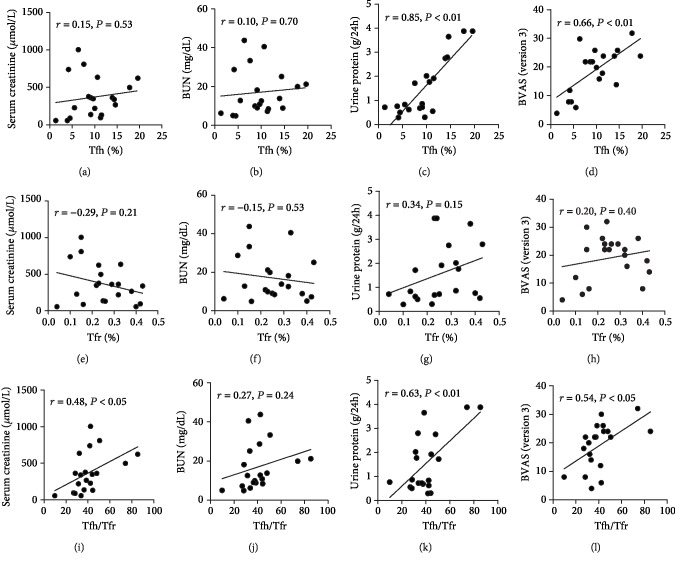
Correlation between circulatory Tfh/Tfr cells and renal injury. Correlation of the percentages of circulatory Tfh cells (a–d), Tfr cells (e–h), and Tfh/Tfr ratios (i–l) with serum creatinine, blood urea nitrogen, 24 h urinary protein levels, and BVAS scores in patients with AAV.

We next evaluated the correlation between Tfh/Tfr ratios and disease activity or renal injury. In patients with AAV, the percentage of circulatory Tfh cells was not significantly correlated with serum creatinine (*r* = 0.15, *P* = 0.53, Figure 2(a)) and blood urea nitrogen (*r* = 0.10, *P* = 0.70; Figure 2(b)) concentrations, but positively correlated with 24 h urinary protein levels (*r* = 0.85, *P* < 0.01; Figure 2(c)) and BVAS scores (*r* = 0.66, *P* < 0.01; Figure 2(d)). The percentage of circulatory Tfr population was significantly correlated with neither serum creatinine (*r* = 0.29, *P* = 0.21; Figure 2(e)), blood urea nitrogen (*r* = ‐0.15, *P* = 0.53; Figure 2(f)), 24 h urinary protein levels (*r* = 0.34, *P* = 0.15; Figure 2(g)), nor BVAS scores (*r* = 0.20, *P* = 0.40; Figure 2(h)). Interestingly, the Tfh/Tfr ratios were positively correlated with serum creatinine (*r* = 0.48, *P* < 0.05; Figure 2(i)), 24 h urinary protein levels (*r* = 0.63, *P* < 0.01; Figure 2(k)), and BVAS scores (*r* = 0.54, *P* < 0.05; Figure 2(l)), but not significantly correlated with blood urea nitrogen (*r* = 0.27, *P* = 0.24; Figure 2(j)).

### 3.4. Increased Circulatory Tfh2 and Decreased Tfh1 Subsets in Patients with AAV

**Figure 3:**
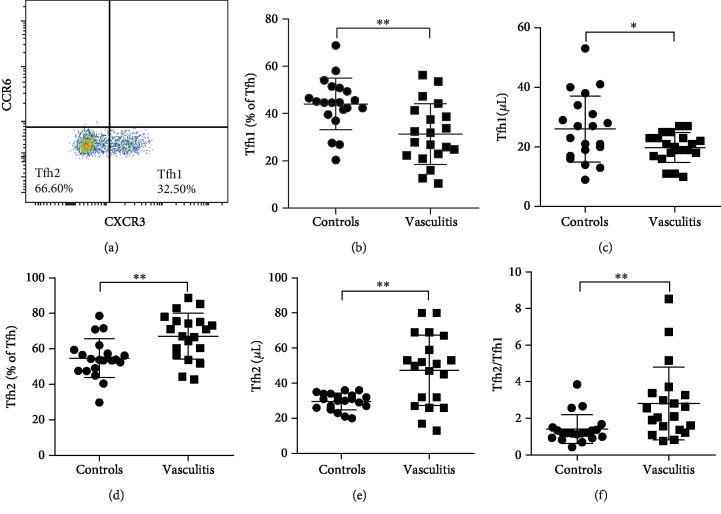
Circulatory Tfh1 and Tfh2 cells in patients with AAV. (a) The gate strategy for circulatory Tfh1 and Tfh2 cells. The percentage and count of circulatory Tfh1 (b, c) and Tfh2 (d, e) cells and Tfh2/Tfh1 ratios (f) in patients with AAV and healthy controls. ^∗^*P* < 0.05; ^∗∗^*P* < 0.01. Tfh: T follicular helper cells.

Tfh cells (CD4^+^CXCR5^+^CD25^−^CD127^interm-hi^) were further divided into Tfh1 (CCR6^−^CXCR3^+^) and Tfh2 (CCR6^−^CXCR3^−^) based on a documented gating strategy [23]. As shown in Figure 3(a), most Tfh cell populations were negative for CCR6, which were defined as Tfh1 or Tfh2. Tfh17 (CCR6^+^CXCR3^−^) and Tfh1/17 (CCR6^+^CXCR3^+^) were too low to be calculated in both patients with AAV and healthy controls. We included the positive control for CCR6 staining in the Supplementary Materials ([Supplementary-material supplementary-material-1]). We found that patients with AAV had a lower percentage and count of circulatory Tfh1 cells while more circulatory Tfh2 cells than healthy controls (*P* < 0.01 or *P* < 0.05, Figures 3(b)–3(e)). In addition, the Tfh2/Tfh1 ratio was increased in patients with AAV when compared to healthy controls (*P* < 0.01, Figure 3(f)). These results suggest that patients with AAV have a shift from Tfh1 to Tfh2 cell populations.

### 3.5. Remission Is Associated with Decreased Tfh Cells

**Figure 4:**
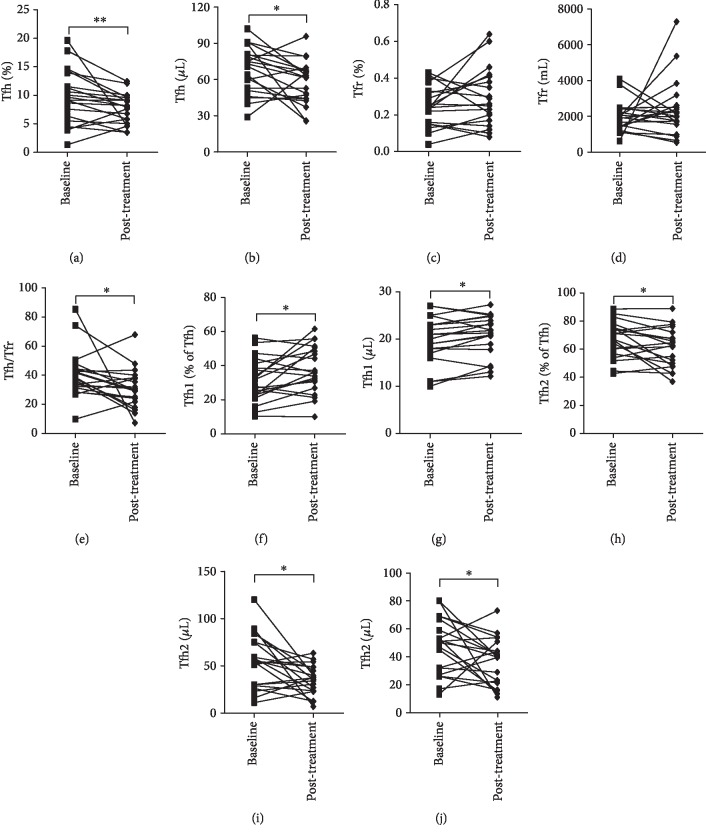
Effects of immunosuppressive treatment on circulatory Tfh/Tfr and Tfh2/Tfh1 ratios. The percentage and count of circulatory Tfh (a, b) and Tfr (c, d) cells and Tfh/Tfr ratios (e) in patients with AAV at baseline and after remission were achieved by immunosuppressive treatment. The percentage of circulatory Tfh1 (f, g) and Tfh2 (h, i) cells and Tfh2/Tfh1 ratios (j) in patients with AAV at baseline and after remission was achieved by immunosuppressive treatment. ^∗^*P* < 0.05; ^∗∗^*P* < 0.01.

Disease remission was reached in all twenty patients with AAV after immunosuppressive treatment with a combination of glucocorticoids and cyclophosphamide. We found that the Tfh cell percentage and count were significantly decreased after the treatment compared to baseline (*P* < 0.01 or *P* < 0.05, Figures 4(a) and 4(b)). However, the Tfr cell percentage and count were not significantly altered by the treatment (Figures 4(c) and 4(d)). Accordingly, the Tfh/Tfr ratios were decreased in AAV patients with remission compared to baseline (*P* < 0.05, Figure 4(e)). In addition, immunosuppressive treatment significantly increased circulatory Tfh1 cell percentage and count (both *P* < 0.05, Figures 4(f) and 4(g)), decreased Tfh2 cells (both *P* < 0.05, Figures 4(h) and 4(i)), and lowered the Tfh2/Tfh1 ratios (*P* < 0.05, Figure 4(j)) in patients with AAV.

### 3.6. Increased Plasma IL-21 Levels in Patients with AAV

**Figure 5:**
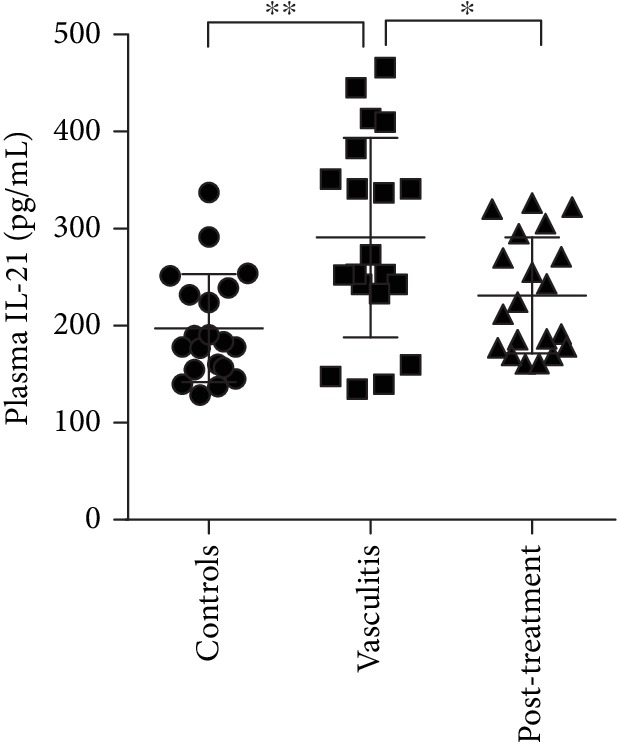
Plasma levels of cytokine IL-21. Plasma IL-21 levels were detected in healthy controls and patients with AAV at baseline and after remission achieved by immunosuppressive treatment. ^∗^*P* < 0.05; ^∗∗^*P* < 0.01.

IL-21 is produced by Tfh cells and is critical for germinal center formation [24]. Plasma IL-21 levels were higher in patients with AAV than in healthy controls (*P* < 0.01, Figure 5). As expected, plasma IL-21 levels were significantly decreased after remission (*P* < 0.05, Figure 5).

## 4. Discussion

Accumulating evidence has shown that dysregulation of Tfh or Tfr cells contributes to the development of autoimmune diseases. Here, we showed that patients with AAV had increased circulatory Tfh/Tfr and Tfh2/Tfh1 ratios and elevated plasma IL-21 levels compared with healthy controls, which were attenuated when disease remission was successfully achieved by immunosuppressive treatment.

Tfh cells are a novel T cell subset in the germinal center [11]. B cells in the germinal center are responsible for producing autoantibodies which are the culprit of a variety of autoimmune diseases, including AAV [5]. Tfh cells play a critical role in autoantibody production through helping B cells form germinal centers and driving B cells to differentiate into antibody-producing plasma cells [25]. Circulatory Tfh cell population is increased in myasthenia gravis, systemic lupus erythematosus (SLE), and multiple sclerosis [26, 27]. The present study identified increased Tfh cells in patients with AAV, which is consistent with previous studies showing that Tfh cells were increased in patients with granulomatosis with polyangiitis and in patients with immunoglobulin A vasculitis [23, 28]. These findings suggest that increased Tfh cells may contribute to the development of AAV.

Tfr cells are a specialized subset of Treg cells [29]. Like Tfh cells, Tfr cells also express CXCR5 which help direct them into the germinal center [14]. Tfr cells play a suppressive role in germinal center reactions [9, 10, 29]. Given that Tfh and Tfr cells have opposite effects on humoral immune responses, the balance between them is critical for immune homeostasis. The uncontrolled decrease of Tfr activity or breakdown of Tfh/Tfr balance can cause autoimmunity, leading to autoimmune diseases [13, 14, 26, 30–32]. For instance, decreased Tfr cells have been found in myasthenia gravis, SLE, and multiple sclerosis [17, 33, 34]. Evidence shows that AAV patients have defective Treg cell function [35]. However, the role of Tfr in AAV has not been reported. The present study demonstrated that patients with AAV had decreased Tfr cells and increased Tfh/Tfr ratios compared with healthy controls. These results suggest that Tfr and Treg cells play a similar role in AAV. Collectively, the findings indicate that imbalance between Tfh and Tfr may contribute to the development of AAV. There are also reports showing increased Treg or Tfr cells in autoimmune diseases, including Sjögren syndrome, SLE, and rheumatoid arthritis [30, 36–40]. These differences may reflect different pathogenic mechanisms between AAV and other autoimmune diseases.

Kidney involvement frequently occurs in patients with AAV, and delayed diagnosis of ANCA-associated renal injury is potentially organ- and life-threatening [41, 42]. In the present study, we found that the percentage of Tfh cells was associated with proteinuria but not serum creatinine and blood urea nitrogen, which suggests that Tfh cells may be related to kidney injury rather than the status of renal function. The percentage of Tfr cells was not associated with either proteinuria or renal function parameters, indicating that Tfr cells may not be a sensitive biomarker for detecting renal injury. Interestingly, the Tfh/Tfr ratio was correlated to both proteinuria and renal function, indicating that the Tfh/Tfr ratio could be a better parameter for evaluating kidney involvement in patients with AAV.

As Tfh cells may play an important role in AAV, we further investigate the distribution of its subtypes. The present study demonstrated that AAV patients had decreased Tfh1 cells, increased Tfh2 cells, and Tfh2/Tfh1 ratios. The results are consistent with those of a previous study showing that Tfh2 cells were increased in child patients with immunoglobulin A vasculitis [23]. However, they found that Tfh17 cells were decreased while Tfh1 and Tfh1/17 cells were not altered when compared to healthy controls [23]. The percentages of Tfh17 and Tfh1/17 were quite low in the subjects of the present study, so these two subsets were not analyzed. Some previous studies also demonstrated that the percentage of Tfh17 was the least subset and was minimal even in healthy controls [34]. The changes and function of Tfh17 cells in autoimmune disease warrant further investigations. Currently, the function of Tfh1 and Tfh2 subpopulations is not well documented. A previous study demonstrated that Tfh1 cells lack the capacity to help naïve B cells and that patients with juvenile dermatomyositis, an autoimmune disease, had decreased Tfh1 and increased Tfh2 cells [43, 44]. These findings suggest that a Tfh1-to-Tfh2 shift might be involved in the development of autoimmune disease. However, a correlation between Tfh1 cells and autoantibody titers was not observed in the present study (data not shown). Taken together, the Tfh2/Tfh1 ratio may be used as a biomarker for AAV, but the role of these subsets in AAV requires further experimental investigation.

We found that immunosuppressive treatment attenuated the increases in Tfh cells and Tfh/Tfr ratios in patients with AAV. These results indicate that Tfh cells and Tfh/Tfr ratios are associated with remission of AAV. These two parameters may be useful for predicting relapse, however, which warrants further study. Previous studies demonstrated that immunosuppressive treatment with sirolimus increased Treg cells [45]. In contrast, the present study showed that remission induction failed to change the decreased Tfr cells, suggesting that immunosuppressive treatment may not target the Tfr subset.

IL-21 is a specific cytokine secreted by Tfh cells. It was not surprising that changes in plasma IL-21 concentrations were consistent with the percentage of Tfh cells. Percentages of IL-21 producing Tfh cells were significantly increased in patients with granulomatosis with polyangiitis [46]. However, plasma IL-21 concentrations in patients with AAV were not reported previously. Given the more feasible measurement of IL-21 than Tfh cells, plasma IL-21 may be a promising biomarker for patients with AAV.

The relationship between Tfh and Tfr cells was not fully understood. Based on the current evidence, Tfh cells may suppress the generation of Tfr cells through secreting IL-21, and IL-21 may play a critical role in the maintenance of Tfh and Tfr cell balance [47]. It was reported that IL-21 was able to suppress Tfr cell-mediated suppressive effects on B cells [48]. In turn, Tfr cells can impair Tfh cells [49]. Taken together, the direct relationship between Tfh and Tfr cells is still unclear. Serial measurements of Tfh and Tfr cells in patients with AAV or *in vitro* experimental studies may help uncover the causative correlation of Tfh and Tfr cells.

There are some limitations in the present study. The sample size was relatively small. The differences in terms of Tfh, Tfr, Tfh1, and Tfh2 subsets between seronegative and seropositive patients could be analyzed with a larger number of cases. The changes of the Tfh/Tfr ratio that we found in the present study may be not specific to patients with AAV. Changes of the Tfh/Tfr ratio may also be present in other autoimmune diseases such as lupus nephritis, which needs to be tested in future studies. Another limitation is that we only selected patients who achieved remission with standard therapy. The comparison between patients with remission and refractory patients should be helpful to define the utility of Tfh/Tfr as a prognostic factor in patients with AAV. In addition, analyzing the relationship between AAV relapse and Tfh/Tfr or IL-21 levels should be helpful in the prediction of AAV relapse.

## 5. Conclusion

In conclusion, circulatory Tfh/Tfr ratios, Tfh2/Tfh1 ratios, and plasma IL-21 levels are increased in patients with AAV and may be associated with disease activity, renal injury, and remission in patients with AAV.

## Figures and Tables

**Table 1:** Demographic and clinical characteristics of the patients.

	Healthy controls *n* = 20	Patients with AAV *n* = 20
Age (years)	64 ± 3	63 ± 2
Sex (females, %)	10 (50.0%)	9 (45.0%)
p-ANCA^+^ (*n*, %)	N/A	16 (80.0%)
c-ANCA^+^ (*n*, %)	N/A	3 (15.0%)
Proteinuria (g/24 h)	N/A	1.56 ± 0.27
Serum creatinine (*μ*mol/L)	72.2 ± 20.3	368.9 ± 60.7^∗∗^
Blood urea nitrogen (mmol/L)	4.1 ± 0.8	17.0 ± 2.6^∗∗^
C-reactive protein (mg/L)		62.1 ± 49.3
Erythrocyte sedimentation rate (mm/h)		65.0 ± 38.1
Diagnosis (*n*, %)		
Granulomatosis with polyangiitis		6 (30%)
Microscopic polyangiitis		14 (70%)
Lung complications (*n*, %)		16 (80%)

^∗∗^ *P* < 0.01*vs.* healthy control.

## Data Availability

The data used to support the findings of this study are available from the corresponding author upon request.

## Supplementary Materials

Supplementary Materials Figure S1: positive controls for CCR6 staining.
