# Supplementary figures and images for: Imbalance of Circulatory T Follicular Helper and T Follicular Regulatory Cells in Patients with ANCA-Associated Vasculitis
Source: Mediators Inflamm. 2019 Dec 2;2019:8421479. doi: 10.1155/2019/8421479 (PMC6914973; doi:10.1155/2019/8421479)

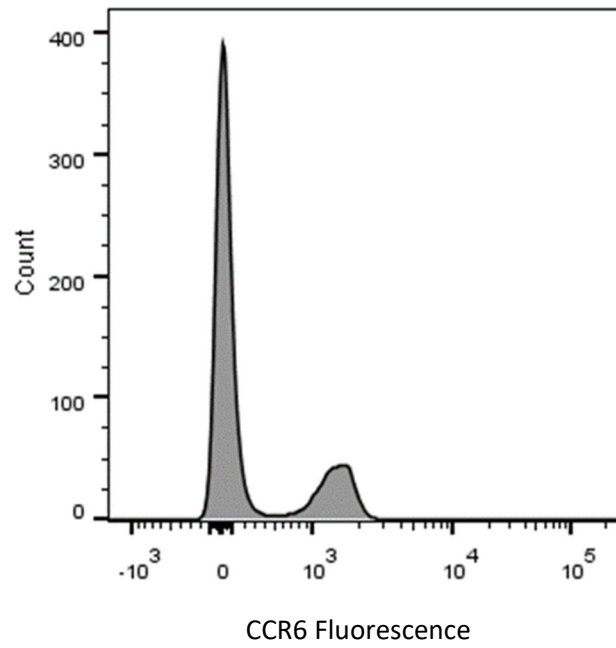

**Figure S1.** Positive controls for CCR6 staining.

Supplement: Supplementary Materials — Figure S1: positive controls for CCR6 staining. [file 8421479.f1.pdf]
